# No evidence for the development of acute analgesic tolerance during and hyperalgesia after prolonged remifentanil administration in mice

**DOI:** 10.1186/1744-8069-9-11

**Published:** 2013-03-07

**Authors:** Hideaki Ishii, Andrey B Petrenko, Tatsuro Kohno, Hiroshi Baba

**Affiliations:** 1 Division of Anesthesiology, Niigata University Graduate School of Medical and Dental Sciences, 1-757 Asahi-machi, Chuo-ku, 951-8510, Niigata, Japan

**Keywords:** Opioid, Remifentanil, Thermal thresholds, Mechanical thresholds, Analgesia, Hyperalgesia, Hypersensitivity, Continuous infusion, Intraperitoneal, Mice

## Abstract

**Background:**

Acute opioid tolerance (AOT) and opioid-induced hyperalgesia (OIH) are undesirable effects of opioids that have been reported in both animals and humans. However, the development of AOT and OIH in cases of potent, short-acting μ-opioid receptor agonist remifentanil administration remains controversial. It has been suggested that the emergence of AOT and OIH by remifentanil could be dose and infusion duration dependent, i.e., low dose and short infusions may lead to negative results. In this study, we determined whether AOT and OIH could be elicited by prolonged, continuous administration of remifentanil at maximally tolerable doses in C57BL/6 mice.

**Results:**

The analgesic effects of continuously administered remifentanil [by short (1 h) and prolonged (4 h) intraperitoneal infusions] were studied. These experiments involved repeated measurements of thermal thresholds during remifentanil administration. Therefore, particular attention was paid to prevent cumulative tissue injury, which could mimic pronociceptive effects of remifentanil. To exclude the possibility of pseudoAOT during infusion, we used brief cooling of all ipsilateral hindpaws that exhibited analgesic response. Thermal thresholds remained steadily elevated over a 1-h period during continuous administration at infusion rates of 120, 180, and 240 mg/kg/h, which indicated no AOT development. To exclude the possibility of pseudoOIH after infusion, intact contralateral hindpaws were used for all postinfusion threshold measurements. Thermal thresholds at each infusion rate returned to the baseline values within 15 min after the termination of the administration. They did not decrease below the baseline values during 1 h following infusion, which indicated no OIH development. Similar threshold dynamics were also observed for thermal and mechanical testing modalities in animals infused at 120 mg/kg/h for 4 h as well as in animals with rapidly attained and maintained maximum analgesia for 3 h.

**Conclusions:**

These results suggest that neither intra-infusion AOT nor postinfusion OIH develops in mice receiving continuous remifentanil when the possibility of cumulative tissue injury mimicking AOT or OIH is carefully avoided.

## Background

Acute opioid tolerance (AOT) is defined as an increase over time of the dose required to maintain adequate analgesia in patients receiving opioid medication for the treatment of pain in clinical settings [1,2]. At the same time, opioid-induced hyperalgesia (OIH) is defined as a state of nociceptive sensitization, which becomes apparent after opioid exposure in clinical settings [1,2]. OIH is characterized by a paradoxical response whereby a patient receiving opioids for pain treatment may have an increased sensitivity to pain.

In animal behavioral studies, AOT is observed as the analgesic potency of an opioid that is rapidly reduced (i.e., within the first several hours) over time. Such reductions can be demonstrated by the attenuation of the potentiating effect of opioids on the immobilizing effects of inhaled anesthetics [3-5]. AOT is described as an analgesic opioid effect that diminishes over time during its administration, whereas OIH is defined as an increased sensitivity to painful stimuli immediately after opioid administration [6,7]. Regarding these pronociceptive opioid effects, some behavioral studies have shown controversial results. For example, one study demonstrated that OIH did not develop immediately following repeated fentanyl injections, whereas nociceptive thresholds were paradoxically decreased on the following day in the fentanyl-treated animals [8]. Another study demonstrated that while prolonged infusion of remifentanil could induce pain hypersensitivity after an infusion (a typical OIH reaction), no reduction of analgesic effect by remifentanil (a typical AOT reaction) was documented during this infusion [9].

Remifentanil, a potent, ultra-short acting μ-opioid receptor agonist, has been gaining popularity in clinical anesthesia. In humans, there are several reports that intraoperative remifentanil can be associated with the development of AOT, which is evidenced by an increased post-operative opioid consumption [10,11]. In contrast, there are also negative reports that intraoperative remifentanil does not cause AOT [12,13]. The existence of AOT and OIH in healthy volunteers remains controversial [14-17].

Several studies have indicated that AOT and OIH could be dose and infusion duration dependent [4,9]. Additionally, it has been suggested that studies reporting negative results using remifentanil could have used low cumulative doses of remifentanil [18].

The purpose of the present study was to clarify the development of AOT and/or OIH with a continuous infusion of remifentanil administered at analgesic and maximally tolerable doses in mice. Although most basic pain research on opioids has been performed in rats, mice were chosen in this study as a research model to evaluate pronociceptive effects of remifentanil because of the availability of mouse transgenic knockouts that can be employed to elucidate the molecular mechanisms of opioid action [19]. This study provided evidence arguing against the existence of AOT and OIH following remifentanil exposure in mice.

## Results

**Figure 1:**
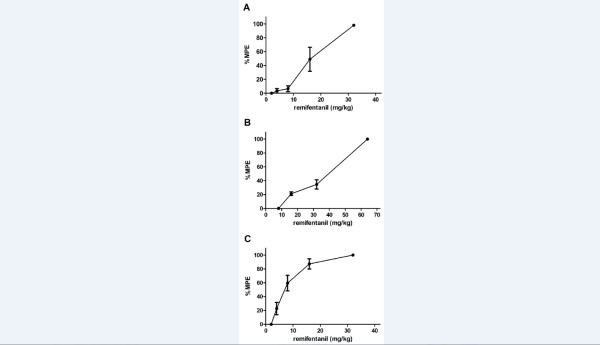
**Dose-effect curves for remifentanil determined by thermal and mechanical nociceptive assays.** (**A**) Hotplate test. The ED_50_ was 16.0 mg/kg (n = 8); the doses were 2, 4, 8, 16, and 32 mg/kg. (**B**) Plantar test. The ED_50_ was 35.7 mg/kg (n = 9); the doses were 8, 16, 32, and 64 mg/kg. (**C**) Randall-Selitto test. The ED_50_ was 6.9 mg/kg (n = 11); the doses were 2, 4, 8, 16, and 32 mg/kg. The data are presented as percent maximal possible effect (%MPE) ± SEM.

Previous studies that have used an intravenous route for remifentanil administration reported analgesic effects when remifentanil was administered in a μg range. However, our preliminary experiments indicated that such doses of remifentanil were ineffective by intraperitoneal administration. Therefore, we performed dose-effect studies by thermal and mechanical nociceptive assays to determine the analgesic potencies of intraperitoneally administered remifentanil, and the results are displayed in Figure 1. The ED_50_ values for the hotplate, plantar, and Randall-Selitto tests were 16.0, 35.7, and 6.9 mg/kg, respectively. Like other opioids, remifentanil also caused behavioral alterations, such as hyperlocomotion and Straub tail reaction (erection of the tail), when administered at doses of 8–32 mg/kg and akinesia and muscular rigidity when administered at doses exceeding 100 mg/kg.

**Figure 2:**
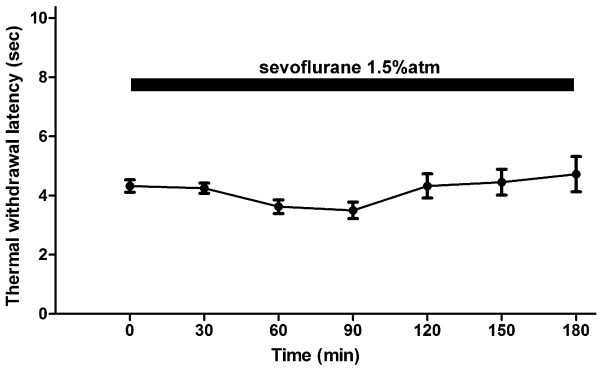
**Thermal thresholds measured by plantar test under sevoflurane anesthesia/sedation.** Four mice inhaled 1.5% atm sevoflurane for 3 h. The latencies did not significantly change throughout the experiment (*P* = 0.2858, one-way ANOVA).

Next, we evaluated the analgesic effect of continuous remifentanil infusion. An infusion was administered via an indwelling intraperitoneal catheter, during which the experiments would normally require animal restraint. However, restraint was avoided by performing the infusion under sevoflurane sedation. To exclude the possibility of sevoflurane (1.5% atm) affecting the sensory thresholds, we concurrently performed a control experiment with no remifentanil in sevoflurane-sedated mice (Figure 2). As indicated in Figure 2, prolonged inhalation of sevoflurane had no effect on the thermal withdrawal thresholds, which remained unchanged for all 3 hours of the experiment (*P* = 0.2858, one-way ANOVA). This finding was consistent with a previous report that indicated no sevoflurane-induced effect on sensory thresholds [7].

**Figure 3:**
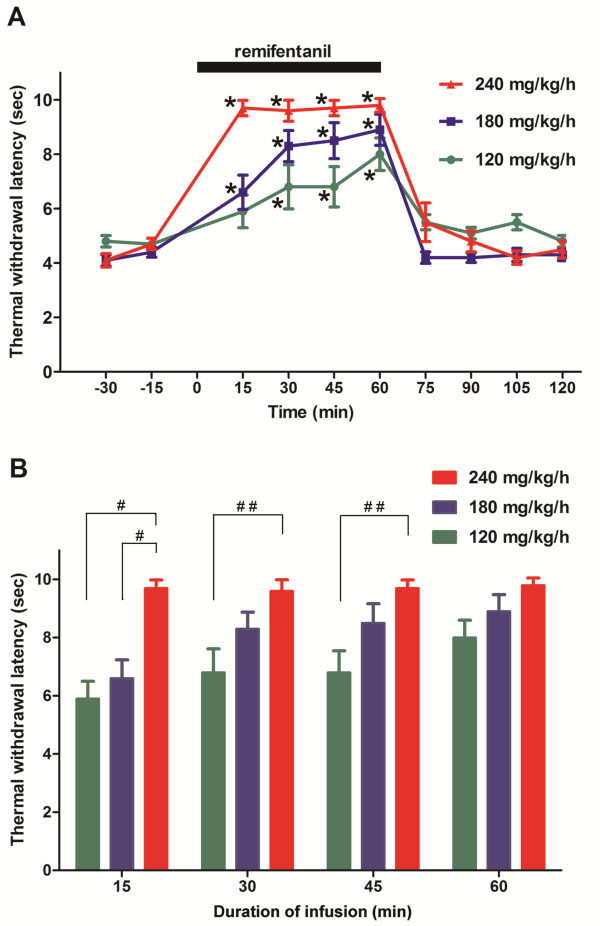
**Effect of short-term (60 min) remifentanil infusions on thermal thresholds measured by plantar test.** (**A**) Time course. The withdrawal latencies increased in a dose-dependent manner. During the infusion, all of the withdrawal latencies were significantly different from the baseline values except at 15 min with an infusion rate of 120 mg/kg/h. (**B**) A comparison of the withdrawal latencies at each time point. At 60 min, the maximum analgesia was reached with all of the infusion rates. The withdrawal latencies at rates of 120 and 180 mg/kg/h were not significantly different from the latency induced by 240 mg/kg/h at 60 min. The data are expressed as the mean ± SEM; n = 8 mice for all of the groups. *P < 0.05 vs. baseline; ^#^*P* < 0.01, ^##^*P* < 0.05 vs. 240 mg/kg/h (one-way ANOVA followed by Dunnett’s test).

The results of the constant-rate infusion experiments that were performed over a 60-min period using the plantar test are shown on Figure 3. Initially, we used a 1-h remifentanil infusion rate that was calculated based on the remifentanil ED_95_ value determined by the plantar test (59.3 mg/kg), and on the duration of a single bolus i.p. injection of remifentanil that did not exceed 15 min (60 mg/kg/15 min × 4 = 240 mg/kg/h). The withdrawal latencies increased in a dose-dependent manner during the remifentanil infusion. A rate of 240 mg/kg/h reached a peak effect approximately 15 min after the start of infusion (Figure 3A). The effects of 180 and 120 mg/kg/h infusions increased gradually and reached their maximum effects at 30 and 60 min after the start of infusion, respectively. The withdrawal latencies at rates of 120 and 180 mg/kg/h were not significantly different from the latency that was induced by 240 mg/kg/h at 60 min (Figure 3B). The withdrawal latencies did not decline during any infusion, which indicated no development of AOT. The analgesic effect of remifentanil was short lasting after terminating the infusion. The latencies for all of the infusion rates returned to the basal values within 15 min after the termination of the infusion and did not decrease below the basal values during the 1-h postinfusion period, which indicated no development of OIH.

**Figure 4:**
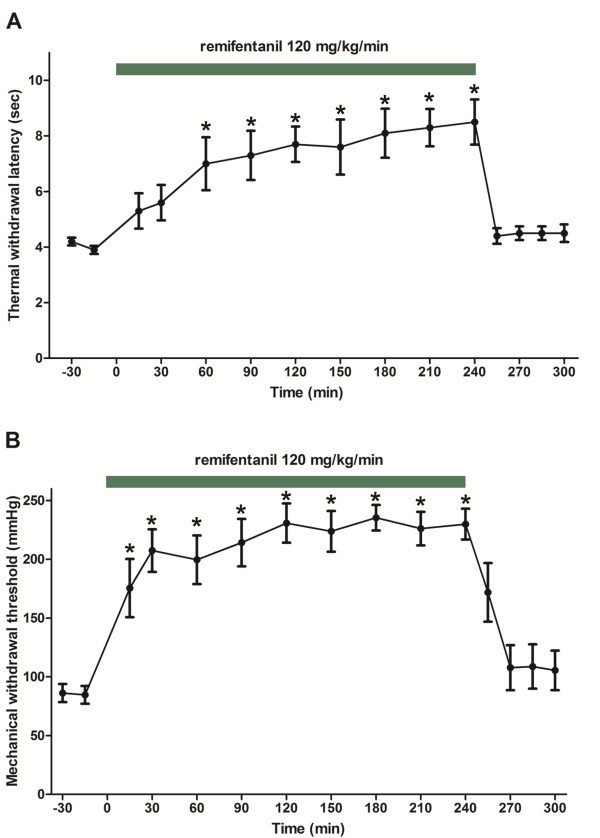
**Effect of a prolonged (4 h) remifentanil infusion at a constant rate of 120 mg/kg/h on nociceptive thresholds.** (**A**) The thermal thresholds measured by plantar test (n = 8). (**B**) The mechanical thresholds determined by Randall-Selitto test (n = 11). The data are presented as the mean ± SEM. *P < 0.05 vs. baseline (one-way ANOVA followed by Dunnett’s test).

Subsequently, we examined the effect of prolonged remifentanil infusions. Preliminary experiments demonstrated that prolonged infusions of remifentanil at rates greater than 120 mg/kg/h could result in the development of muscular rigidity, respiratory depression, and eventually lead to death. The mortality rate at 180 mg/kg/h for 3 h was 50% (6/12). Therefore, to avoid the lethal side effects of prolonged remifentanil administration, we examined the effect of prolonged 120 mg/kg/h remifentanil infusion on thermal and mechanical thresholds (Figure 4). With this infusion rate, the effect of remifentanil increased gradually, and maximum analgesia was reached 1 h after the initiation of infusion. All mice could safely complete the experiment; no lethality was observed. The measured thresholds did not decline during the infusion, which indicated no development of AOT. The measured thresholds returned rapidly to the basal values after completion of this regimen and did not decrease below basal values during the next hour postinfusion, which indicated no development of OIH.

**Figure 5:**
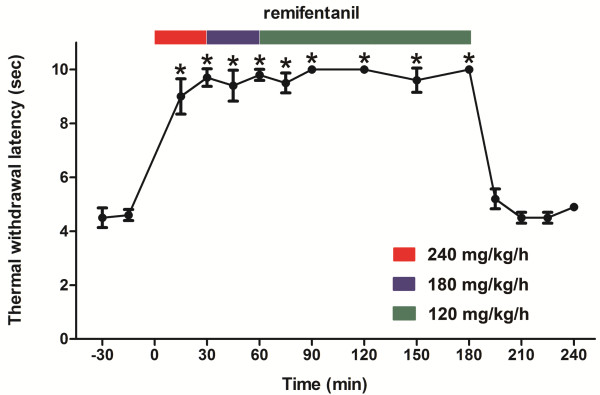
**Effect of a prolonged (3 h) and tapered remifentanil infusion on thermal thresholds measured by plantar test.** The infusion was started at a rate of 240 mg/kg/h that was reduced to 180 mg/kg/h at 30 min and continued at 120 mg/kg/h at 60 min until the end of the infusion. The data are expressed as the mean ± SEM, n = 6. *P < 0.05 vs. baseline (one-way ANOVA followed by Dunnett’s test).

In another experiment, to rapidly attain and maintain analgesia at a maximum level while avoiding lethality, we examined the effect of a tapered remifentanil infusion on thermal thresholds. As shown in Figure 5, this infusion was initiated at 240 mg/kg/h for 30 min and subsequently reduced to 180 mg/kg/h for 30 min, with a final rate of 120 mg/kg/h that was maintained for 2 h. The maximum effect could be maintained for 3 h without causing lethality. With this regimen, the measured thresholds remained elevated during the infusion and did not decrease below the baseline levels after the termination of the infusion, which indicated no development of either AOT or OIH even when the maximal analgesic effect of remifentanil was sustained for 3 h.

## Discussion

Although there are numerous reports of the existence of AOT and OIH in rodents, human studies have produced conflicting results. It is unclear what accounts for the equivocal results in humans or the differences between humans and animals. Several articles have reviewed the methodological and theoretical issues with respect to discrepancies [2,18,20]. It has been suggested that methodological issues could lead to conflicting results. These issues, which could lead to a crucial misinterpretation, were administration dose and duration. There is also a possibility of artificially caused hyperalgesia by the repetitive and potentially tissue-damaging nature of the stimuli used to determine the threshold during opioid infusion.

The administration dose of this study should have been more than sufficient to elicit AOT and OIH. We found that with the intraperitoneal route, attaining analgesia by remifentanil required infusion rates in the range of mg/kg/h. This is in contrast to several other studies that reported analgesia by remifentanil when administered in μg/kg/h range by the intravenous route [3-5]. The intraperitoneal injection of remifentanil may require higher doses because of the esterase-dependent metabolism of this drug. Remifentanil is rapidly metabolized by non-specific blood and tissue esterases. The hydrolysis of remifentanil by esterases appears to occur at a limited rate in blood and more rapidly in tissues. In fact, Davis et al. reported that tissue metabolism was the main factor in remifentanil hydrolysis [21]. Because the majority of remifentanil is metabolized in tissues, its intraperitoneal injection and immediate, direct exposure to tissue esterases might require significantly higher doses of remifentanil compared with that of an intravenous injection. The possibility of accelerated local tissue cleavage of remifentanil, a carboxylic acid methyl ester, upon its intraperitoneal administration is also supported by the fact that mesothelial cells of the peritoneum are known to contain high levels of carboxylesterase [22].

In this study, we selected an alternative parenteral route of remifentanil administration via a catheter placed in the intraperitoneal space, whereas most animal studies of remifentanil analgesia used rats and an intravenous route of drug administration. This selection was due to technical difficulties related to tail vein catheterization in mice weighing < 30 g. As it was previously mentioned, although this route required unconventionally high remifentanil doses, such doses were necessary and also sufficient to elicit powerful analgesic effect, which occurred for single bolus injections at postinjection times comparable with other anesthetic agents administered via this route [23-25]. During the 4-h 120 mg/kg/h infusions, the development of a robust analgesic effect against thermal stimuli required certain time, whereas an analgesic effect was quite rapidly attained against mechanical stimuli. This could be because of the more damaging nature to living organisms of thermal stimuli compared with mechanical ones. Additionally, in a separate set of experiments, we used a tapered remifentanil infusion to rapidly attain maximum analgesic effect of remifentanil against thermal stimuli. However, even in this setting, neither AOT nor OIH was observed. Thus, it appears unlikely that a somewhat “slow” intraperitoneal route *per se* could have precluded the development of AOT or OIH in our study. In fact, some studies that have reported acute pronociceptive effects of remifentanil have utilized an even slower subcutaneous route for its continuous infusion [7,26].

The duration of the remifentanil administration also should have been sufficient to elicit pronociceptive effects of remifentanil should they develop. AOT in animal studies is typically investigated during continuous infusion over 2–3 h, whereas OIH is usually assessed within 1 h postinfusion [3-6,9,27]. Consequently, the same parameters were adopted as those previously reported: remifentanil was infused continuously for 3–4 h, and the effects were investigated for 60 min postinfusion in the current study. However, in this study, despite a sufficient duration and full analgesic effect of the remifentanil infusion, we could not document the development of AOT or OIH.

Remifentanil infusions were performed under sedation with 1.5% sevoflurane, and the possibility of this anesthetic preventing the development of AOT or OIH should be considered. This appears to be relevant because one study has previously suggested that sevoflurane can prevent the development of hyperalgesia in rats that received four repeated subcutaneous injections of 60 μg/kg fentanyl [28]. However, the possibility of sevoflurane affecting the results of our study is unlikely, because a prevailing number of reports have demonstrated the development of pronociceptive effects of remifentanil in rats or mice despite the fact that they were inhaling sevoflurane at concentrations comparable or even greater than those used in this study [3-5,7,26,29,30]. The unlikely remifentanil-sevoflurane interaction is further supported by the fact that, compared with other inhalational anesthetics, sevoflurane has only a minimal inhibitory effect on NMDA receptors [31], which are considered to be involved in the development of opioid-related hypersensitivity [32].

When nociceptive thresholds are repeatedly measured in a situation in which protective withdrawal reflexes are impaired or abolished by opioid administration, the possibility of cumulative tissue injury manifesting as AOT or OIH should be carefully excluded [33]. In our experience, even limiting exposure to heat by cutoff points set at ≥2-fold the threshold (e.g., 10 sec vs. baseline of ≈ 4 sec as was performed here) if left unattended can result in thermal injury. This is especially likely to occur with repetitive testing protocols. Additionally, with the testing-during-the-infusion protocols, a longer opioid infusion is equivalent to more injury to the stimulated site. Comparison with saline controls in such a situation would be inadequate because withdrawal responses in the absence of opioids are preserved throughout the experiment, meaning that no injury is inflicted.

What differentiated our study from other studies that reported pronociceptive effects of remifentanil is the careful attention and exclusion of the possibility of such cumulative tissue damage, which could occur due to repetitive nociceptive testing if performed under the opioid cover. In our study, tissue damage as a possible cause of AOT and OIH was carefully avoided. Thermal tissue injury, which could have been misinterpreted as AOT during the infusion, was avoided by cooling of the ipsilateral hindpaws after each threshold determination indicative of analgesic response (See Methods section). Any residual thermal or mechanical tissue damage, which could have manifested after the infusion in the ipsilateral hindpaws as OIH, was avoided by using the contralateral hindpaws for postinfusion measurements. Using contralateral hindpaws was possible because of the systemic nature of OIH that, if it develops, can be detected at any stimulated site of the body. Thus, having carefully excluded the possibility of artificially induced and opioid-unrelated increase in pain sensitivity, we have found no evidence for the development of remifentanil-induced pronociceptive effects in our experimental mouse model.

## Conclusions

Continuous infusion of remifentanil did not induce AOT and OIH as evidenced by a steady analgesic effect of remifentanil during infusion and the uneventful normalization of nociceptive thresholds after the termination of its infusion. Our results in mice support most human data that indicate no development of AOT and OIH after remifentanil administration. These findings also indicate a need for critical reevaluation of experimental protocols that are aimed to examine pronociceptive effects of opioids and involve repetitive thermal testing during opioid administration in animals.

## Methods

### Animals

This study was approved by the Animal Research Committee of the Niigata University Graduate School of Medical and Dental Sciences in Niigata, Japan. All of the procedures were performed on adult (8–14 weeks of age) male C57BL/6 mice, weighing 17–22 g at 8 weeks. The animals were housed in cages (4–5 mice per cage) under a standard 12-h light/dark cycle with food and water available *ad libitum*. The temperature of the testing room was kept at 24 ± 2°C, and the experiments were conducted between 09:00 and 18:00. All of the experiments except the hotplate test were performed under light sevoflurane anesthesia/sedation. The mice used in the hotplate experiment were habituated to the testing room environment for 1 h (including 15 min of placing the mice on the turned-off hotplate apparatus) for three consecutive days before the test to become quiescent and allow for reliable data collection [24]. All of the mice were used in several experiments and were left undisturbed for ≥1 week after each experiment to provide sufficient time for recovery. In each experimental session, one stimulus modality (thermal or mechanical) and one dosing regimen were used. All efforts were made to minimize animal suffering and the number of animals used.

### Drugs

Remifentanil (Ultiva®) and sevoflurane (Sevofrane®) were purchased from Janssen Pharmaceutical (Tokyo, Japan) and Abbott Japan (Tokyo, Japan), respectively. Remifentanil was dissolved in normal saline (0.9% NaCl).

### Determination of analgesic range of intraperitoneally administered remifentanil

The analgesic effects of single bolus intraperitoneal injections of remifentanil were evaluated by measuring the thermal and mechanical pain thresholds by hotplate, plantar, and Randall-Selitto tests. Remifentanil was injected in a volume of 2.5 ml/kg using a 30-gauge needle after an aspiration test. For the hotplate test, the doses were 2, 4, 8, 16, and 32 mg/kg. For the plantar test, the doses were 8, 16, 32, and 64 mg/kg. For the Randall-Selitto test, the doses were 2, 4, 8, 16, and 32 mg/kg. The preliminary experiments established that the analgesic effect of remifentanil first became evident approximately 2 min postinjection, and 3–4 min postinjection was a time after which the maximum response to intraperitoneal remifentanil was achieved. After determining the basal latencies, thermal and mechanical latencies were measured 3 min after each dose injection and at least 15 min were allowed before the injection of the next dose. The analgesic data are presented as the percentage of maximal possible effect (%MPE) according to the following formula [34]:

$$\begin{array}{cc} \;\%\text{MPE} & =\left(\text{post}-\text{drug}\;\text{threshold}–\text{pre}-\text{drug}\;\text{threshold}\right)/\\ & \;\left(\text{cutoff}\;\text{threshold}–\text{pre}-\text{drug}\;\text{threshold}\right)\times 100\% \end{array}$$

The 50% analgesic doses (ED_50_) were calculated by performing nonlinear regression analyses.

### Continuous infusion of remifentanil

Analgesic effects of continuous remifentanil administration on thermal and mechanical thresholds were studied during short (1 h) and prolonged (3 or 4 h) remifentanil infusions. For this purpose, the mice were individually placed in an induction chamber into which 5% atm sevoflurane in a continuous oxygen flow of 1 L/min was administered. After induction, the mice were transferred to individual small plastic chambers that were connected to a vaporizer and 100% oxygen source as described previously [23]. The concentrations of sevoflurane were continually monitored using an infrared gas analyzer (Capnomac Ultima, Datex Instrumentarium, Helsinki, Finland). Rectal temperatures were determined using a digital thermometer (TD-300, Shibaura Electronics, Saitama, Japan). A plastic plate that was filled with circulating hot water and placed below the chambers was used to actively maintain the body temperature between 36–38°C. The mice were kept under spontaneous ventilation during the entire experiment.

The implantation of an intraperitoneal catheter for continuous infusion was performed under 3% atm sevoflurane. The abdominal wall was gently lifted, and a 20-gauge Tuohy needle (Perifix, B Braun, Tokyo, Japan) was inserted in the mid abdomen after disinfection with chlorhexidine and carefully advanced for approximately 1 cm. A catheter for continuous infusion was introduced through the needle for approximately 2 cm into the intraperitoneal cavity. After removal of the needle, the catheter was fixed to the skin with 5–0 silk suture.

All threshold determinations, including those before, during, and after the infusions, were performed under sevoflurane administered at a sedative concentration of 1.5% atm. After a 15-min equilibration period for sevoflurane, the intraperitoneal administration of remifentanil was started and kept constant at 0.2 ml/h by an infusion pump (FP-1000, Melquest, Toyama, Japan). The thermal and mechanical thresholds were determined on both hindpaws before the infusion, on the ipsilateral hindpaws during the infusion, and on the contralateral hindpaws after the infusion. During the infusions, repeated brief cooling of thermally stimulated ipsilateral hindpaws was used to avoid the cumulative thermal injury that could mimic AOT (see Behavioral Testing: Thermal sensitivity). Additionally, to exclude a possibility of any residual thermal injury mimicking OIH after the infusion, contralateral hindpaws were chosen for postinfusion thermal threshold measurements. During preliminary experiments, repetitive mechanical stimulation was shown to be less damaging and did not result in decreased thresholds on the ipsilateral side postinfusion compared with thermal stimuli. However, for reasons of better experimental reliability, postinfusion measurements of mechanical thresholds were also performed on contralateral hindpaws. The baseline thermal and mechanical thresholds were averaged bilateral latencies that were determined 30 and 15 min prior to the commencement of the infusion (0 min). The latencies were measured every 15 or 30 min during and after the infusion for short-term and prolonged infusions, respectively.

### Behavioral testing: thermal sensitivity

Thermal sensitivity was evaluated by a hotplate test in conscious mice and a plantar test in sedated mice. An apparatus consisting of an acrylic cage and temperature-controlled plate was used for the hotplate test (Hotplate analgesia meter, IITC/Life Science Instruments, Woodland Hills, CA, USA). The mice were placed on a 52°C hotplate, and reaction times to lick the hindpaw or jump were recorded. A maximum cutoff time of 60 sec was used in the absence of a response.

The plantar test was conducted using a Hargreaves plantar apparatus (Ugo Basile Inc., Varese, Italy) [35]. During the test, only the hindpaws were exposed while the mice were kept in the anesthetic chambers. Thermal sensitivity was assessed by measuring hindpaw withdrawal latencies to a radiant heat stimulus. The time until the withdrawal of the hindpaw was automatically recorded. A maximum cutoff time of 10 s was used in the absence of a response. Our preliminary experiments demonstrated that repetitive thermal stimulation performed during a 180 mg/kg/h 1-h remifentanil infusion when protective withdrawal reflexes were absent because of opioid analgesic effect could cause cumulative burn injury that was evident by erythematous changes in the plantar skin and paw swelling and manifested as hyperalgesia (average 1-h postinfusion latency of 2.7 ± 0.1, n = 6, vs. an average baseline latency of 4.6 ± 0.8, n = 6, *P* = 0.0210, paired *t*-test). Therefore, in subsequent remifentanil experiments, for all analgesic responses defined for quick practical reference as latencies >6 s, we cooled the stimulated hindpaws in ice-cold water for 2× the measured latency to avoid cumulative burn injuries during the infusion. The beneficial effects of immediate cooling on the burn wounds have been well recognized in the literature [36-40]. In this study, the absence of thermal injury in the stimulated ipsilateral hindpaws was confirmed by their careful visual inspection throughout the experiment, during and after the remifentanil infusion, and on the following day. The postinfusion measurements were performed in the intact contralateral hindpaws without cooling.

### Behavioral testing: mechanical sensitivity

Mechanical withdrawal reflexes were evaluated by the Randall-Selitto test in sedated mice [41]. Mechanical pressure was applied to the hindpaws using a pressure analgesia meter (MK-201D, Muromachi Kikai, Tokyo, Japan). The application of pressure continued until the mouse withdrew its hindpaw or the pressure reached 250 mmHg, which was established as a cutoff pressure in the absence of a response.

### Statistical analysis

Statistical analysis was performed by GraphPad Prism software version 5.04 (GraphPad Software Inc., San Diego, CA, USA). The responses at each time point during the prolonged remifentanil infusions were compared with baseline measures using repeated-measures one-way ANOVA with Dunnett’s posttest. The differences between the three remifentanil doses at different time points during the 1-h infusion were analyzed by one-way ANOVA followed by Dunnett’s posttest. All of the data are presented as the mean ± SEM. *P* < 0.05 was considered statistically significant.

## Competing interests

The authors have no financial or other relationship that could lead to a conflict of interest.

## Authors’ contributions

HI, ABP, TK, and HB designed the experiments, and HI, ABP performed the experiments and analysis. HI, ABP, TK, and HB prepared the manuscript. All authors read and approved the final manuscript.
